# Optogenetics and Targeted Gene Therapy for Retinal Diseases: Unravelling the Fundamentals, Applications, and Future Perspectives

**DOI:** 10.3390/jcm13144224

**Published:** 2024-07-19

**Authors:** Merve Kulbay, Nicolas Tuli, Arjin Akdag, Shigufa Kahn Ali, Cynthia X. Qian

**Affiliations:** 1 Department of Ophthalmology & Visual Sciences, McGill University, Montreal, QC H4A 3S5, Canada; merve.kulbay@mail.mcgill.ca; 2 Faculty of Medicine and Health Sciences, McGill University, Montreal, QC H3G 2M1, Canadaarjin.akdag@mail.mcgill.ca (A.A.); 3 Centre de Recherche de l’Hôpital Maisonneuve-Rosemont, Université de Montréal, Montreal, QC H1T 2M4, Canada; shigufa.kahn.ali.cemtl@ssss.gouv.qc.ca; 4 Department of Ophthalmology, Centre Universitaire d’Ophtalmologie (CUO), Hôpital Maisonneuve-Rosemont, Université de Montréal, Montreal, QC H1T 2M4, Canada

**Keywords:** optogenetics, targeted gene therapy, inherited retinal diseases

## Abstract

With a common aim of restoring physiological function of defective cells, optogenetics and targeted gene therapies have shown great clinical potential and novelty in the branch of personalized medicine and inherited retinal diseases (IRDs). The basis of optogenetics aims to bypass defective photoreceptors by introducing opsins with light-sensing capabilities. In contrast, targeted gene therapies, such as methods based on CRISPR-Cas9 and RNA interference with noncoding RNAs (i.e., microRNA, small interfering RNA, short hairpin RNA), consists of inducing normal gene or protein expression into affected cells. Having partially leveraged the challenges limiting their prompt introduction into the clinical practice (i.e., engineering, cell or tissue delivery capabilities), it is crucial to deepen the fields of knowledge applied to optogenetics and targeted gene therapy. The aim of this in-depth and novel literature review is to explain the fundamentals and applications of optogenetics and targeted gene therapies, while providing decision-making arguments for ophthalmologists. First, we review the biomolecular principles and engineering steps involved in optogenetics and the targeted gene therapies mentioned above by bringing a focus on the specific vectors and molecules for cell signalization. The importance of vector choice and engineering methods are discussed. Second, we summarize the ongoing clinical trials and most recent discoveries for optogenetics and targeted gene therapies for IRDs. Finally, we then discuss the limits and current challenges of each novel therapy. We aim to provide for the first time scientific-based explanations for clinicians to justify the specificity of each therapy for one disease, which can help improve clinical decision-making tasks.

## 1. Introduction

Inherited retinal diseases (IRDs) encompass a group of genetically complex diseases that lead to gradual vision loss and blindness, with a prevalence of 1 case in 3000 individuals worldwide [1]. A recent study using computational analysis estimated that approximately 2.2 billion individuals worldwide are carriers of an IRD, from which 5.5 million individuals are expected to have clinical manifestations [2]. Furthermore, the financial burden associated with the diagnosis and treatment of IRDs is substantial in North America; costs attributable to IRDs in the United States was shown to range from nearly USD 13,500 million to USD 32,000 million [3]. Loss of wellbeing, followed by loss of productivity, were the major contributors to these expenses [3]. Consequently, there is a growing urge to develop novel treatments, such as gene therapies, to limit the progression of vision loss and the decrease in vision-related quality of life.

Numerous classification systems can be employed to categorize IRDs, such as the inheritance pattern, the clinical course of the course (i.e., stationary vs. progressive), based on the anatomical and functional disease features, or the phenotypic and genotypic characteristics [4,5]. A recent comparative cross-sectional study proposed a novel classification system based on the latter method, dividing the retinal dystrophies into six distinct categories, consisting of diffuse photoreceptor dystrophies, macular dystrophies, chorioretinal dystrophies, inner retinal and vitreoretinal dystrophies, systemic diseases associated with photoreceptor dystrophies, and congenital and stationary retinal diseases [5].

The genetic mutations involved in the pathogenesis of IRD have been shown to lead to the loss or degradation of the photoreceptor layer or retinal pigment epithelium (RPE) [6]. These mutations are inherited through the conventional inheritance patterns (i.e., autosomal dominant (AD), autosomal recessive (AR), X-linked, and mitochondrial inheritance patterns) [6]. Multiple studies have underscored the genetic epidemiology behind IRDs [7,8,9,10,11,12]; a recent study from Karali and colleagues identified by next-generation sequencing more than 1300 sequence variations in 132 genes, which included 866 potential genotypes for therapeutic avenues [7]. Gene therapy and optogenetics have generated tremendous interest over the past years to tackle the treatment of retinal dystrophies [13,14,15,16,17]. Herein, we review the advantages of these novel systems for the management of IRDs, the fundamental concepts involved in their engineering, and the most recent preclinical and clinical studies. 

## 2. Optogenetics and Targeted Gene Therapies: Novel Advances in the Treatment of IRDs

**Figure 1:**
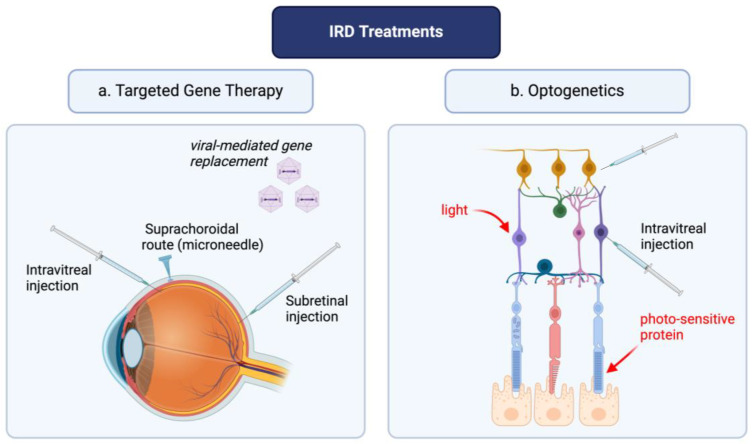
Optogenetics and targeted gene therapy: a novel approach for inherited retinal disease management. The figure was created with BioRender.com.

Current treatment of retinal dystrophies involves a multidisciplinary approach, given the limited clinical tools to halt disease progression in many disease subtypes [18]. The role of reactive oxygen species (ROS) in the pathogenesis of retinal diseases has been thoroughly described in previous studies and comprehensive literature reviews [19,20]. Briefly, the pro-oxidative environment ensuing gene mutations leads to photoreceptor degeneration, retinal ganglion cell death and retinal remodeling characterized by modulations in gene expression, migration of retinal cells, and neovascular modifications [21]. To delay disease progression in IRD, such as RP, multiple solutions have been proposed such as the use of antioxidants; in vivo studies have shown great potential of these agents in delaying disease progression [22,23]. However, challenges remain in the treatment of patients with gene mutations; the progression of the disease is inevitable and ultimately leads to blindness. Gene therapy and optogenetics are modern medicine tools whose primary aim is to treat genetic disorders at the level of the dysfunctional retinal cells (Figure 1). However, the engineering of these latter tools requires a multi-step approach, which is discussed in the ensuing sections.

## 3. Optogenetics

The concept of optogenetics emerged in the early 1980s, with the premise that light modulation could target and control specific neurons [24]. Throughout the years, scientific breakthroughs shaped the current modern concept of optogenetics, which aims to restore visual function by integrating opsins (i.e., light-sensitive proteins) into the retinal neurons. This process aims to control the activity of opsin-transfected cells by modulating light exposure/excitation. Prior to delving into the molecular principles involved in optogenetics, it is crucial to understand physiologic phototransduction. 

### 3.1. Phototransduction

**Figure 2:**
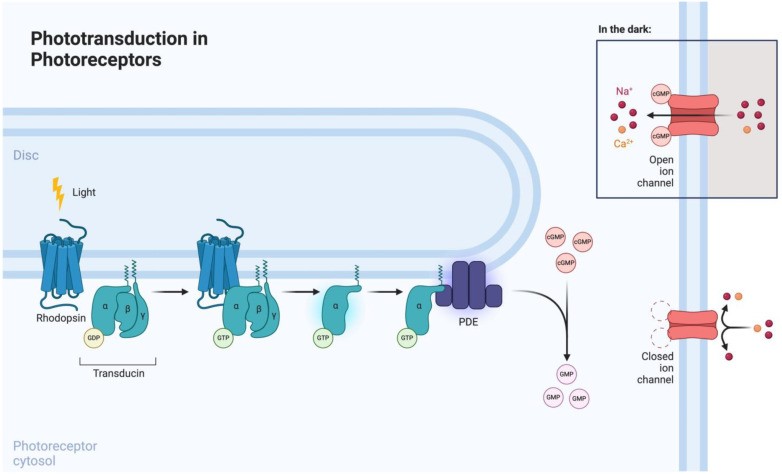
Phototransduction signaling pathway in photoreceptors. Reprinted from “Phototransdution in Photoreceptors” by Biorender.com (2024). Retrieved from https://app.biorender.com/biorender-templates (accessed on 1 June 2024).

Phototransduction is mediated by photoreceptors (i.e., rods and cones), which are highly specialized neurons involved in visual function (Figure 2). Rods are involved in scotopic vision (i.e., vision at low levels of ambient light), whereas cones mediate photopic vision (i.e., vision at higher levels of ambient light) [25]. Both cell types consist of a highly organized anatomical structure, which encompasses an elongated outer segment, cilium, inner segment, cell body, and terminal synapse [26]. Rhodopsin, a rod-specific opsin and G-protein-coupled receptor (GPCR), is the key mediator of phototransduction, which takes place in the outer segment. Upon light exposure, rhodopsin undergoes photoisomerization, leading to the formation of its active form, metarhodopsin. Metarhodopsin activation subsequently induces transducin recruitment and intracellular GTP-mediated phosphodiesterase (PDE) signaling [27]. Therefore, by inducing the expression of opsins in degenerated photoreceptors, it is possible to modulate the phototransduction signaling pathway.

### 3.2. Optogenetic Engineering 

To achieve an efficient optogenetic system, three main components are required: an opsin, a vector for transfection, and a safe surgical approach for drug delivery. Opsins form the backbone of optogenetics. In the ensuing section, we review the main categories of opsins and their advantages and disadvantages in optogenetics. The vector of transfection and surgical approaches are discussed in further sections. 

#### Opsins

**Figure 3:**
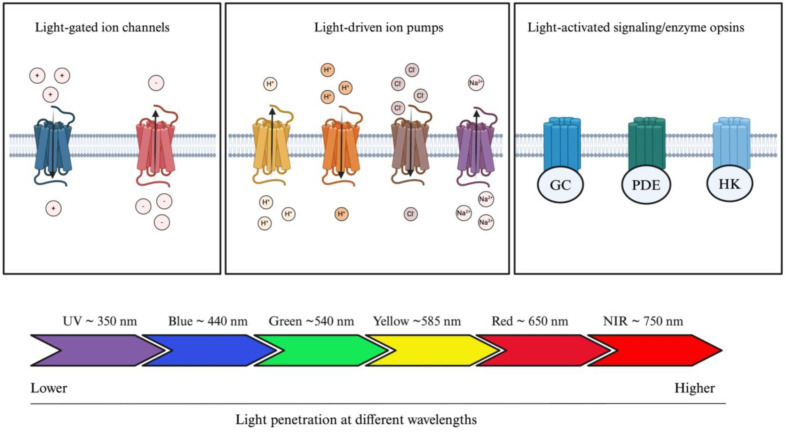
Optogenetic actuators and their tissue-penetrating properties. Optogenetic actuators can be divided into three distinct groups: light-gated ion channels, light-driven ion pumps, and light-activated signaling/enzyme opsins. Light source can be modulated according to clinical application; blue light is the least penetrating, whereas red light penetrates the most human tissue. Abbreviations: GC, guanylyl cyclase; PDE, phosphodiesterase; HK, histidine kinase; UV, ultraviolet; NIR, near infrared. The figure was created with BioRender.com.

Opsins are G-protein-coupled receptors (GPCRs) and can be divided into two categories: microbial (also known as type 1 opsins, consisting of prokaryotes, algae, and fungi) or animal opsins (also known as type II opsins) [28]. Microbial opsins have been mostly studied for the treatment of IRD and can further be divided in three distinct subcategories, which consist of light-gated channels, light-driven ion pumps, and light-activated signaling/enzyme opsins (Figure 3) [29]. Microbial opsins use light as a substrate. Photostimulation induces opsin-mediated activation, silencing, or signaling pathway modulation of photoreceptors [30]. They function by absorbing light at a specific wavelength. Greater wavelengths are known to be associated with greater penetration rates (i.e., penetrating deeper into the skin) [31]. Blue light is the least penetrating at a wavelength of approximately 440 nm (peak sensitivity), whereas red light travels further given its approximate 650 nm wavelength (peak sensitivity) [32]. This phenomenon is partly explained by the different light scattering properties and absorption coefficients in human tissues [32,33]. Therefore, by modulating the wavelength properties of opsins, it is possible to modulate tissue penetration and opsin delivery within the human body. Given the greater penetrating ability of wavelengths greater than 650 nm, red or NIR wavelengths are preferred in optogenetic engineering, which is further discussed.

Light-gated channels encompass cation or anion channel rhodopsins (ChRs) [29]. These ChRs can be subdivided into further categories based on their maximal wavelength sensitivity: blue-opsins (i.e., opsins stimulated by blue light) or red-opsins (i.e., opsins stimulated by red-light). ChR2, an activating cation light-gated channel, is the most widely studied microbial rhodopsin throughout the literature. ChR2, derived from *Chlamydomonas reinardtii*, mediates the transport of Na^+^, K^+^, H^+^, and Ca^2+^ ions across the photoreceptor cell membrane following blue light stimulation and subsequent depolarization [34]. It is capable of further depolarizing the retinal ganglion cells (GCs), initiating electrical signals that replicate the role of photoreceptor cells. The objective of this method is to reinstate light sensitivity and facilitate the transmission of visual information to the brain. However, given the low absorption rate of blue light opsins, ChR2 is not a great candidate for deep tissue penetration, such as the RPE [29]. Furthermore, it was shown that blue light opsins require greater energy levels to be stimulated, which can further damage the retina or further aggravate the IRD [35,36]. To leverage these challenges, red opsins have shown greater potential in optogenetics (Table 1). However, an additional challenge with red opsins is their non-negligeable blue light sensitivity, which can lead to cross-talking in dual color systems. Chimeric opsins, as detailed in Table 1, can be used to minimize the cross-talk induced by red opsins. The signaling pathway of chimeric opsins can be divided into a two-step process [37]. First, the red opsins are inactivated by utilizing a long red stimulus. In the second step, immediately following the inactivation of red opsins, blue opsins are stimulated. This process allows for the exclusive stimulation of blue opsins, while red opsins are fully inactivated.

Light-driven ion pumps allow the unidirectional transport of single cations or anions across the cytoplasmic membrane. They can be subdivided into four distinct categories, consisting of outward or inward proton pumps, chloride pumps, and sodium pumps. Bacteriorhodopsin (BR) was the first ever characterized light-driven outward proton pump [38]. Halorhodopsin (HR) and archaerhodopsin (Archs) are also light-driven ion pumps [39]. The most recent discoveries involves the HR family opsins, with studies developing variants of *Np*HR [40,41]. Finally, the last category of opsins encompasses light-activated signaling/enzyme opsins, where intracellular signaling pathways can be modulated with light. Sensory rhodopsins (SR) were the first opsins from their category to be characterized. However, with recent advances, three novel opsins have been characterized, consisting of histidine kinase rhodopsin (HKR), guanylate cyclase rhodopsin (Rh-GC), and rhodopsin-phosphodiesterase (Rh-PDE) [42,43,44,45], which are all transmembrane proteins with eight domains. Overall, the evolving library of mutant microbial opsins is significantly contributing to the extending range of application of optogenetics and renders its suitability for various pathogenic mechanisms.

**Table 1:** Summary of current and potential microbial opsins for optogenetics.

Category	Opsin Examples ^a^	Advantages	Disadvantages	References
Light-gated ion channels				
Blue light opsins	ChR2 *Gt*ACRe	Rapid cell depolarization following stimulation (<50 μs)	Limited tissue penetration Requires high stimulus intensity in comparison to physiological rhodopsin and cones	[16,35,36,46]
Red-shift opsins	VChR1 ReaChR Chrimson ChrimsonR ChrimsonSA ChRmine frChRmine	Greater tissue penetration	Non-negligeable blue-light sensitivity that may lead to cross-activation	[47,48,49,50,51]
Chimeric opsins	Chronos/ChrimsonR CheRiff/ChrimsonR ChR2/ReaChR ChR2/ChrimsonR	Highly specific modulation of red or blue opsins	Low population of blue opsins can limit the excitatory potential	[52,53,54,55,56,57,58,59,60,61]
Light-driven ion pumps				
Hydrogen pumps	BR Arch Mac	Production of higher photocurrent rates and less interference with neurotransmission		[62,63]
Sodium pumps	KR2 (DeNaR)	Efficient for neuron silencing		[64,65]
Chloride pumps	HR NpHR e*Np*HR 2.0 e*Np*HR 3.0	Rapid activation and inactivation kinetics Efficient for neuron silencing	Low levels of generated photocurrent with *Np*HR Requires high stimulus intensity in comparison to physiological rhodopsin and cones, as well as ChR2	[66,67,68,69]
Light-activated signaling/enzyme opsins				
Sensory rhodopsins (SR)	SRI SRII			
	HKR Rh-GC Rh-PDE	Selective modulation of intracellular signaling pathways		[42,43,44,45]

^a^ Abbreviations: ChR, channelrhodopsin; BR, bacteriorhodopsin; Arch, archaerhodopsin-3; Mac, Leptosphaeria maculans; KR; Krokinobactereikastus rhodopsin; HR, halorhodopsin; NpHR, Natromonas pharaonis halorhodopsin; SR, sensory rhodopsin; HKR, histidine kinase rhodopsin; Rh-GC, guanylyl cyclase rhodopsin; Rh-PDE, phosphodiesterase rhodopsin.

## 4. Genome Editing with CRISPR-Cas9

Genome editing is a promising therapeutic strategy to correct underlying mutations that underly IRDs. First-generation genome editors, which include zinc-finger nucleases (ZNFs) and transcription-activator like effector nucleases (TALENs), have been studied extensively but with limited success [70,71]. Both are endonucleases with modifiable domains that can introduce double-strand DNA breaks (DSBs) at specific loci. However, they have been limited by their complexity [72]. More recently, genome editing has focused on the clustered regularly interspaced short palindromic repeats (CRISPR)—the CRISPR-associated protein 9 (Cas9) system. CRISPR-Cas9 system is composed of nucleases discovered in bacteria, in which they function as an adaptative immune system protecting prokaryotes against bacteriophage and plasmid infection [73]. CRISPR-Cas9 is more promising than first-generation genome editors due to its relative simplicity, adaptability, and specificity [72,74,75]. 

In bacteria, CRISPR systems rely on two small RNAs that detect foreign pathogenic nucleic acids and guide their endogenous Cas protein to the infecting nucleic material and cleave it, thus protecting the host [73]. Three types (I-III) of CRISPR systems have been discovered and studied. In 2012, a protocol for describing CRISPR-Cas9 as a genome editing tool was first published, and since then, it has been investigated as a therapeutic tool for IRDs [73,76,77]. For genome editing, the RNAs are merged into a single, chimeric guide RNA (gRNA), combining the functions of both prokaryote RNAs. The gRNA must contain a protospacer adjacent motif (PAM) sequence and an accompanying 20 nucleotide target sequence, which will guide the Cas9 protein to the target site where Cas9 will introduce a double-strand break (DSB) [77]. This DSB will be repaired by cellular mechanisms such as non-homologous end-joining (NHEJ) or homology-directed repair (HDR). Thus, unlike TALENs and ZFNs, which require modification of the nuclease, CRISPR-Cas9 is much simpler as its selectivity is achieved by adjusting the gRNA and not the Cas9 endonuclease. 

### System Engineering

**Figure 4:**
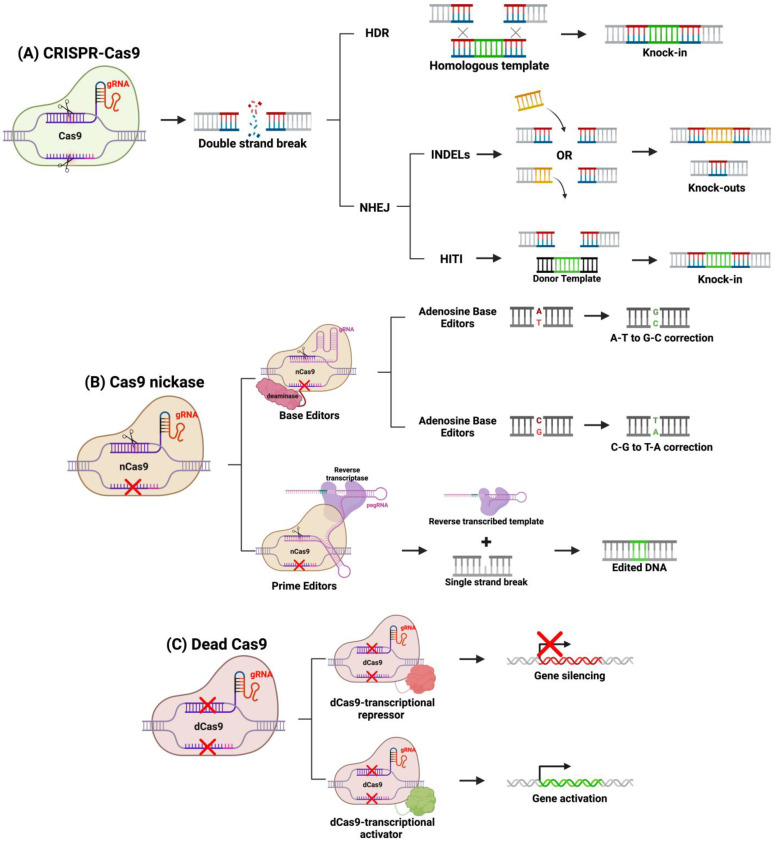
CRISPR-based gene editing methods. (**A**) Using a fully functional system, CRISPR-Cas9 will target a specific sequence by the gRNA and will cause a DSB. Knock-ins can be achieved through HDR or HITI mechanisms, whereas knockouts are achieved through NHEJ indels. (**B**) Cas9 nickases can be linked to deaminases (base editors) or reverse transcriptase (prime editors) to precisely correct mutations. (**C**) Dead Cas9 can be linked to transcription factors to alter gene expression. Figure created with BioRender.com.

Over the past decade, genome editing applications of CRISPR-Cas9 have been expanded, and various methods have been developed to modify the system to edit genes in diverse ways. For example, DSBs using a traditional Cas9 protein favor NHEJ or HDR repair, and through protocol design, a specific DNA repair pathway can be activated over the other (Figure 4). NHEJ introduces small insertions and deletions (indels) disrupting protein function. This repair pathway is active throughout the cell cycle in all dividing and non-dividing cells, making it ideal in the quiescent retina [77,78,79]. NHEJ is useful to knock-out gain-of-function or dominant-negative mutations such as Rhodopsin (*Rho*) gene mutations in autosomal dominant retinitis pigmentosa (ADRP) [78,79]. NHEJ can be leveraged to restore normal expression through a “reduction and replacement” strategy. This strategy involves reducing the expression of the mutated gene product, then transfecting a wild-type gene, thus restoring phototransduction signaling pathway [80]. This method is ideal in restoring normal protein expression in the presence of a toxic mutant. Another group expanded upon the “reduction and replacement” strategy by developing a method called homology-independent targeted integration (HITI), where the “replacement” transgene is integrated at the site of the Cas9 DSB [72,79,81]. By designing the DNA template to contain a homologous cleavage site as the gene-of-interest, the researchers demonstrated that integration is achieved through NHEJ [81]. This is achieved by Cas9 cleaving both the template and target gene, and NHEJ will repair both breaks by integrating the template into the genome. HITI can be a preferred strategy compared to “reduction and replacement” strategy because the complementary DNA (cDNA) is integrated into the host genome at its endogenous locus, ensuring long-term expression in dividing cells and endogenous gene regulation. Homology-directed repair (HDR) is another cellular DNA repair mechanism that is activated in response to DSB introduced by Cas9 and requires a homologous template like HITI. In the presence of a homologous sequence, HDR will integrate the sequence into the Cas9 cleavage site. Therapeutically, it can be designed to correct single-nucleotide or monoallelic mutations involved in IRDs. HDR-based CRISPR genome editing was shown to correct small 5 bp deletions or a point mutation in mouse models of IRDs [82,83]. However, HDR-based CRISPR-therapeutics may not be the most effective option for IRDs, as HDR is active primarily in mitotically active cells [72,79]. Since most cells of the retina are post-mitotic, HDR will be downregulated in the retina, and thus NHEJ-based CRISPR therapies are more suited for gene editing for IRDs [72,79]. 

Newer developments in CRISPR therapeutics have modified the Cas9 protein by altering its cleaving function. Several of these strategies now exist, one of which is fusing catalytically inactivated or “dead” Cas9 (dCas9) to transcriptional regulators. Silencing the expression of this toxic products from mutated genes in IRD can be achieved by fusing a dCas9 to a transcriptional repressor and targeting this construct to the mutant allele with a specific gRNA [84]. As a result, only the dominantly expressed toxic allele is repressed, restoring normal rhodopsin signaling. Alternatively, dCas9 can be fused to transcriptional activators to upregulate silent genes to replace loss-of-function mutants [85]. Many genes have counterparts with homologous function, but in certain cell types, only one of such genes is expressed. For example, in rhodopsin-deficient mouse models of RP, there is a lack of rhodopsin-dependent signaling in rods [85,86]. In cones, M-opsin provides a homologous function to rhodopsin, but it is silenced in rods. Thus, by transfecting cells with a transcriptional activator of M-opsin fused to dCas9, M-opsin can be transactivated in rods, restoring their photon-sensitive signaling, which will improve retinal function and attenuate retinal degeneration [85]. dCas9 allows the targeting of transcriptional regulators to specific genomic sites, opening the possibility of therapeutically altering gene expression instead of modifying DNA sequence. 

dCas9 are generated by inactivating Cas9 catalytic sites. Alternatively, Cas9 can be mutated to inactivate only one catalytic site generating Cas 9 nickases (Cas9n) that only cleave one DNA strand [87,88]. New editing tools, called DNA base editors (BEs) and prime editors (PEs), are composed of Cas9n fused to a deaminase or reverse transcriptase, respectively. The enzyme and Cas9n fusion protein can precisely correct single-point mutations at the site of a single strand break [87,88]. BEs are further categorized into cytidine deaminase (cytosine BE or CBE) or deoxyadenosine deaminase (adenosine BE or ABE) [87,88]. The CBE or ABE will be guided by a gRNA to a target sequence, where CBEs deaminate a C-G base pair to a U-G base pair, or ABEs deaminate a T-A base pair to an I-T base pairs. Cas9n will then cleave the non-deaminated strand, creating a single-strand break that will be repaired by host mechanisms and resolve the point mutation [88]. The overall nucleotide edit is a C to T transition by CBEs and an A to G transition by ABEs, and a more recent protocol has expanded this system to generate C to G transitions using C to G BEs (CGBEs) [87,88,89]. This strategy can be extremely promising for resolving mutations that underly several IRDs [90].

Unlike BEs, PEs possess the potential to repair both point mutations and transcribe short sequences that can be integrated through host DNA repair mechanisms [91]. PEs have a unique gRNA termed a prime editing guide RNA (pegRNA), which directs the PE to a specific locus and provides the template for reverse transcriptase. Once the nickase makes a single-strand break, the template transcribed by the reverse transcriptase is integrated into the host genome at the target locus through host repair mechanisms [91,92]. The utilization of reverse transcription allows for the correction of any transition or transverse mutations, enabling precise correction of IRD-causing point mutations with minimal off-target edits [79,93]. Furthermore, unlike BEs, PEs can also correct small indels. A limitation to PEs is their large size, which hampers its delivery via adeno-associated virus (AAV) vectors and therefore may limit the effectiveness of this strategy [91]. These CRISPR-Cas9 strategies demonstrate the promise of the Cas9 in treating IRDs. Long-term success of these strategies would rely on their safety and efficacy within the retina. Importantly, one study in Cas9 knock-in mice evaluated the long-term effects of Cas9 within the eye [94]. This study demonstrated the maintenance of the structural and functional components of both the retina and RPE, which is key pre-clinical evidence demonstrating the promise of Cas9-based strategies in long-term treatment of IRDs [94].

## 5. RNA Interference for IRDs

Our genome is constantly being transcribed into mRNA that is then translated to protein. mRNA is processed and spliced prior to translation, which serves as a second wave of regulation to ensure proper protein expression. The time between transcription and translation where mRNA is processed, modified, and spliced is a window of opportunity for RNA interference (RNAi) therapies to alter gene expression without having to act at the gene level.

While mRNA is being transcribed, our genome is simultaneously transcribing non-coding RNA (ncRNA), which are not translated into protein but serve as mRNA regulators. They play an important functional role in the retina, regulating factors in several cellular pathways that when dysregulated can lead to disease. As such, ncRNAs and their role in regulating RNAs can be central to disease pathophysiology and can be leveraged therapeutically. RNAi is a growing therapeutic field based on ncRNA, which include micro-RNA (miRNAs), small interfering RNA (siRNA), short hairpin RNA (shRNA), and antisense oligonucleotides (ASOs) (Table 2).

### System Engineering

miRNAs are short nucleic acids, typically 20–25 base pairs, that are endogenously expressed in human genomes. They are transcribed in the nucleus as single primary miRNA and later cleaved by nuclear and cytosolic RNAses II, Drosha and Dicer [95]. The final product of this enzymatic activity is a double-stranded miRNA that binds argonaute (AGO) proteins and forms RNA-induced silencing complexes (RISC) [95]. Within RISCs, miRNAs serve as guides, homing this complex to an mRNA target complimentary to the miRNA. Once the miRNA anneals to its target, the RISC complex interferes with translation or activates mRNA degradation. This strategy can be potentiated, as one miRNA can target hundreds of endogenous mRNA genes, highlighting its therapeutic potential [95,99]. Therapeutic miRNA can be delivered to the retina in AAV vectors through subretinal injections to slow down retinal degeneration in IRD mouse models [97,98]. 

siRNAs are 21–23 bp, double-stranded RNAs that were first discovered in plants and later in mammalian cells, where they protect host cells from viral pathogens [96]. Therapeutically, siRNAs are activated intracellularly by cytoplasmic Dicer that cleaves long double-stranded pre-siRNA molecules [95]. Similar to miRNA, siRNAs are incorporated into RISC complexes and serve as a guide to target host mRNA for degradation [95]. Furthermore, a single siRNA can target multiple mRNAs, as they are recycled for multiple rounds of mRNA cleavage, similar to miRNA [95]. Early studies in IRDs had limited success due to a lack of stability and efficiency of siRNAs [100,101]. However, a recent study demonstrated that intravitreal injections of chemically modified siRNA termed tetra-valent siRNA (tetra-siRNA) in mouse and pig retinas could effectively and safely silence their target [101]. Despite the promise of such modifications, siRNAs studied to treat age-related macular degeneration have been shown to trigger immune responses, as non-internalized siRNA trigger extracellular receptors that initiate immune cascades causing retinal degeneration [102]. Both these studies demonstrate the promise and current limitations of siRNA-therapies in silencing toxic mutant expression in IRDs [101,102].

shRNAs are 19–22 base pair, double-stranded RNA connected by a 4–11 base pair hairpin loop that integrates into RISC complexes and silences or degrades specific mRNA, similar to miRNA and siRNA. However, they are unique as they are delivered in an exogenous DNA expression vector, typically an AAV vector, and not as an RNA effector molecule. This strategy allows for sustainable long-term expression by the host cells. This strategy is favorable, especially in the quiescent retina, as it eliminates the requirement of continuous administration, which is the case for miRNA and siRNA [95]. In ADRP, shRNA has been shown to be functionalized as a “knockdown and replace” strategy similar to CRISPR methods previously outlined [95,103]. 

ASOs are a class of single-stranded DNA or RNA molecules, typically 15–30 base pairs in length. Functionally, they interfere with mRNA translation by silencing, degrading, or altering splicing of their target. Silencing is achieved by designing an ASO that will interfere with ribosomal binding to the target mRNA. Degradation occurs when ASOs are developed to recruit RNases to degrade mRNA. Finally, splicing can be manipulated by designing ASOs that target defective splice sites to restore normal splicing. In clinical use, ASOs have been limited by nuclease degradation within cells, but modification of chemical modification of ASOs, forming phosphonodiamidite morpholino oligonucleotides (PMOs), can improve delivery by reducing degradation [104].

## 6. Vectors for Optogenetics and Targeted Gene Therapy 

Gene therapies and optogenetic systems are most efficiently introduced to target cells through vectors, which maximize transduction efficiency, while ensuring that therapies with multiple constructs such as CRISPR, composed of Cas9 and gRNAs, transduce cells concurrently (Table 3). These vectors can be injected by different routes (i.e., subretinal, intravitreal, and suprachoroidal), allowing efficient transduction into photoreceptors and the RPE. 

The first vectors studied for gene therapy were adenoviruses (AdVs). AdVs, despite being very efficient transducers, caused significant inflammation, which has limited their use as therapeutic vectors [105]. AAVs have since replaced AdVs. They belong to the family of parvoviruses and are reliant on co-infection, primarily of AdVs, for replication. Their genome is quite simple, containing a single strand of DNA of about 4.8 kb, which is surrounded by a protein shell that will interact with carbohydrates on the surface of target cells to transfect cells [107]. 

AAVs have been the preferred vector for genetic editing and have become popular for several reasons. The first is that AAVs have a low integration rate into the host genome, eliminating the possibility of integration-related mutations that can cause loss-of-function mutations or activate oncogenes. Secondly, AAVs are very efficient at delivering constructs due to their high diffusion and transduction capacity, especially in quiescent retinal cells [108]. Furthermore, AAVs are non-pathogenic helper-dependent viruses that require co-infection to replicate, reducing the possibility of activation. Finally, various serotypes exist (AAV1-13), and combining these serotypes to create pseudotypes can further refine delivery [105]. For example, a common pseudotype is AAV2/8, which contains AAV2 genome in an AVV8 capsid [105]. Although they are the preferred vector in gene-editing studies in IRDs, there are some key limitations that have led to the investigation in other vectors. Of note, AAVs are immunogenic. However, most importantly in the context of IRD gene therapy, AAVs are limited by their small packaging size. With a genome of 4.8 kb, they can hardly accommodate Cas9. For CRISPR-based therapeutics, dual AAV systems are frequently used, which limits transduction efficiency, as both AAVs need to transduce the same cell for the desired effect [108]. This limited size is also insufficient to treat certain IRDs. Specifically, the most frequently mutated gene in Usher syndrome, *MYO7A* (7 kb) [110]*,* and in Stargardt disease, *ABCA4* (128 kb) [111]*,* exceed the carrying capacity of AAVs. However, for most other IRDs, such as RP, LCA, and Cone-rod dystrophy, AAV or dual-AAV systems have been shown to effectively transduce with CRISPR and RNAi construct in preclinical models and clinical trials [81,112]. Conversely, AAVs have also shown great efficacy and safety for optogenetic system delivery [113,114].

Due to the limited packaging size of AAVs, lentiviruses (LVs) have been studied for gene therapy for diseases such as Stargardt disease and Usher syndrome that require larger packaging size. LVs can transduce large genes, as their carrying capacity is almost double that of AAVs, with a genome of around 8 kb [109]. LVs were first derived from HIV-1, but these had poor transduction of photoreceptors, and improved transduction has been demonstrated with equine infectious-anemia-virus-derived (EIAV) LVs [109]. LVs are limited by several factors. First, they are more likely to cause an immune response compared to AAVs [105]. More importantly, however, LVs naturally integrate into the host genome, which yields long-lasting expression, but also poses a safety concern due to insertional mutagenesis that can activate oncogenes [105]. Importantly, modifications can be made to LVs to reduce their integration capacity [105]. LVs are used in IRD studies, specifically for gene therapies for Stargardt disease and Usher syndrome and may be preferred to delivering large CRISPR constructs such as PEs [108,109]. Conversely, in optogenetics, lentiviral platforms, such as the OPTO-BLUE and Light-On systems, have been successfully used to induce light-controlled expression of reporter proteins [115,116].

Nanoparticles are the newest vectors that have shown immense promise in gene therapy delivery in other organs. Most notably, lipid-based cationic nanoparticles were used in COVID-19 vaccines [108]. Compared to viral vectors, they are easy to produce at scale in liquid form, and they can be chemically modified or incorporate different ligands to alter transduction efficiency based on cell target [109]. Nanoparticles also have a better safety profile as they are less immunogenic and possess less insertional mutagenesis risk [108]. Furthermore, given their lower invasive nature, they induce lesser levels of inflammation and limit tissue damage [117]. Finally, there are diverse subtypes of nanoparticles of varying sizes that can incorporate large constructs, a key limitation to AAVs [108,109]. Despite current limited use of these vectors in IRD clinical trials, they are expected to be the future vectors of gene therapy for IRDs. Some early studies have demonstrated some promising results. One study demonstrated that intravitreal injections of PEGylated-ECO nanoparticle carrying plasmid *ABCA4*-DNA was shown to be safe and effective in mouse models of Stargardt disease [118]. Furthermore, using upconversion nanoparticles, researchers were able to use near-infrared (NIR) light sources to activate optogenetic proteins [119,120,121]. Overall, the possibility to deliver nanoparticles through an intravitreal injection would be a clear advantage over viral vectors that require subretinal injections, a much more complicated procedure. However, it is to note that other non-viral delivery methods have been explored for the delivery of optogenetics constructs, such as the use of electroporation [122] and biopolymers (e.g., hydrogels) [123,124]. Given the clinical significance and importance of AAVs in drug delivery, we mainly focus on these delivery systems in the ensuing section.

## 7. Recent Advances in Optogenetics

Among the range of innovative approaches for vision restoration, optogenetic therapy stands out for its ability to confer light sensitivity onto remaining retinal neurons through the introduction of ectopic light-responsive proteins [125]. This technique can prove therapeutic to a wider range of patients as it can treat the disease independently to the underlying gene defect. A wide array of optogenetic actuators have been utilized paired to various promoters to explore their potential in restoring vision. This section aims to elucidate the extent to which meaningful improvement in vision can be achieved by modulating retinal ganglion cells (RGCs), bipolar cells (BCs), or photoreceptors with optogenetic actuators. Herein, we provide a summary of the recent periclinal studies published within the last five years (Table 4).

### 7.1. Delivery of Optogenetic Actuators to Retinal Ganglion Cells

Despite the loss of photoreceptor cells in many cases of IRD, the remaining retinal layers, including RGCs, often remain intact and maintain communication with the brain through the optic nerve, providing an avenue for stimulation to potentially restore vision. Targeting RGCs could potentially treat patients, regardless of disease stage, with loss of all photoreceptors. 

Recent advancements are aimed at developing a ChR with improved channel kinetics that is more photosensitive with the goal of restoring daylight vision [133]. This resulted in the creation of a modified ChR by replacing the amino acid sequences related to ion-conduction in mVChR1 with ChR2 counterparts. This chimeric opsin, ex3mV1CO, has shown greater sensitivity compared to mVChR1 [133]. Additionally, VEPs were recorded 17 months after transfection. Apart from ChR2, the application of other light-sensitive proteins with absorption spectra shifted towards the red end of the spectrum for optogenetic treatments of IRD has been explored. ReaCh or CrimsonR respond to longer wavelengths of light, such as red or NIR. The use of these red-shifted opsins holds promise in enhancing light sensitivity in patients by reaching deeper into the retina. With the goal of more specific opsin targeting to the membrane, Gauvain et al. optimized an AAV2.7m8-ChR-tDT vector [128]. Upon single IV injection of the ChrimsonR construct, primates were found to have an estimated restored visual acuity of 20/249 based on MEA recordings. In contrast to mice and other non-human mammals, primates possess a fovea similar to that of humans, rendering them optimal subjects for in vivo studies of vision restoration. McGregor et al. used IV delivery of ChrimsonR and the calcium sensor GCaMP6s, using a dual AAV2 vector and in vivo imaging to demonstrate optogenetic responses of RGCs in non-human primates [131]. They found that the ChrimsonR mediated optogenetic responses of inner retinal neurons, which persisted 14 months after IV injection. Furthermore, additional variants for ChR2, such as CoChR, were optimized in terms of light sensitivity and kinetics by an increase in the deactivation time [132]. 

A recent preclinical study utilized Chronos, a blue channel rhodopsin [129]. Chronos is reported to be tenfold more light-sensitive than ChR2, threefold more than ChrimsonR, with a longer excitation wavelength (with peak excitation at 500 nm) with fast on/off kinetics resulting in a substantial decrease in the risk of potential phototoxic effects [48]. By estimating the spatial resolution of the retina, a recent study on nonhuman primates concluded that targeting ganglion has the potential to yield visual acuity surpassing the threshold for legal blindness [130]. Ferrari et al. injected macaque retinas with an AAV2 encoding CatCh (human codon optimized ChR variant bearing L132C mutation [143]) under a RCG-specific promoter. They used a classical linear-non-linear (LN) model for the CatCh reactivated macaque retina to simulate the spiking response of the reactivated retina to an acuity test (the random E test) and performed Bayesian decoding at different time points following stimulation to predict quantitatively the best visual acuity one can expect in a patient. Based on the spatial resolution of the retinas, their model predicted that a patient should be able to discriminate letters corresponding to a visual acuity of 20/72. In contrast to epiretinal implants, which have been shown to activate distant ganglion cells and thus limit vision restoration, the reactivated GCs in the transduced primate retina were only sensitive to the stimulation of their dendritic field and soma. 

Microbial opsins are limited as to their low light sensitivity or slow kinetics due to the lack of signal amplification. Additionally, these opsins lack adaptation to changes in natural light [134]. Conversely, type 2 opsins frequently attach covalently to 11-cis-retinal, initiating metabotropic signaling that indirectly affect ion channels upon light exposure. Furthermore, in the mammalian retina, it is expected that animal opsins would elicit a diminished immunogenic response [144]. A novel approach to confer light sensitivity involves the utilization of cone opsins, specifically the vertebrate middle wave opsin (MW-opsin) [134]. However, it is limited in its latency as its off-response time is >10 s [135]. In two sets of behavioral tests, *rd1* mice expressing the MW-opsin displayed a pronounced inclination toward the dark compartment during a light avoidance task. Additionally, these mice demonstrated exploratory behavior, specifically an ability to differentiate between constant and pulsating light, discern moving lines of varying spatial frequencies, and investigate unfamiliar objects across diverse natural light environments. Electrophysiological assessments conducted in the V1 region revealed enhanced stimulus detection capabilities, with responses adapting to fluctuations in brightness. Operationally, this therapy can prove suitable in both indoor and outdoor light levels, circumventing the need for intensifying goggles. Visual restoration was similarly achieved with the introduction of a Gleobacter/human chimeric rhodopsin (coGHCR) in a murine model [139]. To study the functional outcome of different optogenetic targets, a direct comparison of AAV-mediated expression of CoChR in ON-BP cells versus RGCs was performed using a TKO mouse model (Opn4^−/−^ Gnat1^−/−^ Cnga3^−/−^) [138]. MEA recordings from the retina showed that the threshold light intensity to elicit a spike potential in RGCs was 1 log unit lower (2.0 × 10^13^ photons/cm^2^/s) compared to bipolar cells (2.4 × 10^14^ photons/cm^2^/s) [138]. Additionally, when comparing RGC targeting to BC targeting at equivalent light intensity, the RGCs targeted transfections demonstrated higher visual acuity [138]. Furthermore, significant pupil constriction was observed in TKO mice with RGC expression, not in those where BCs were targeted [138]. Thus, the authors concluded there is a higher efficacy of restored vision when targeting RGC compared to ON BC. However, in recent investigations, both animal-derived opsins (hOPN4) and microbial opsins (ReaChR) were targeted to bipolar cells and retinal ganglion cells (RGCs) to assess and contrast their response times and sensitivity. They concluded that bipolar targeted optogenetic tools exhibited higher light sensitivity and faster kinetics when compared to RGC targets [145]. Thus, there is no unanimous agreement regarding the favored cell type for targeting.

### 7.2. Delivery of Optogenetic Actuators to Bipolar Cells

As cell loss is mainly restricted to the outer retina in IRDs, BCs also remain mainly intact even in later stage disease [137]. Specifically targeting BCs is thought to better mimic the intrinsic processing features of the retinal circuitry, providing ON and OFF responses at the downstream RGCs. Unlike RGCs, BCs lack lateral extensions, resulting in a more focal activation pattern [135]. Additionally, when directly imparting light upon RGCs, one bypasses the parallel presynaptic processing of visual information such as luminance, directed movement, and contrast done by bipolar and amacrine networks. A BC-targeted approached would thus be an ideal method to preserve inner retinal processing [136]. However, downstream retinal neurons are affected by significant remodeling at end-stage RD compared to RGCs.

Studies have shown the potential of optogenetic actuator delivery to BCs. Injection of MCO1, a multi-characteristic, highly photosensitive opsin targeted onto ON BC of *rd10* mice, resulted in stable expression up to 4 months after delivery [135]. Additionally, MCO1 has a broad spectral response, allowing for vision restoration in multiple color environments [146]. Significant improved visually guided behavioral outcomes showing light sensitivity were quantified through water maze and optomotor assays. Notably, this engineered opsin showed improved optomotor response at ambient light levels <10 mW*/mm^2^ [135]. Furthermore, Gaub et al. targeted retinal ON-bipolar cells of rd1 mice with a rhodopsin construct under control of the 4xgrm6 promoter. MEA recordings showed robust responses with an amplitude akin to WT across a broad spectrum of light strengths of treated retinas [147]. Firing rates were similar to wild-type retinas. Additionally, the results of behavioral tests showed restoration of innate light avoidance and temporal pattern recognition. The treated mice were able to distinguish between light and dark as well as between static and moving spatial patterns. Similarly, Kralik et al. transducted a modified GPCR construct comprising of the transmembrane region of melanopsin paired with the intracellular segment specific to ON-bipolar cells found in mGluR6 into the retina of *rd1* mice. This engineered opsin restored cortical light responses. By activating the mGluR6 signaling cascade, these chimeric opsins were demonstrated to be 3–4 log units more sensitive than microbial alternatives [148]. 

### 7.3. Delivery of Optogenetic Actuators to Photoreceptors

Few studies have further demonstrated the clinical significance of optogenetic actuators in photoreceptor function modulation [69,141]. However, even following optogenetic therapy, cones are likely to continue on a degenerative path. Thus, this method is not optimal for patients with advanced disease where there is only a narrow window to target cones.

### 7.4. Clinical Trials

The first clinical trials of optogenetic treatment for IRDs are currently underway (Table 5). Various companies and research institutions are making notable progress in clinical trials.

For instance, RetroSense Therapeutics, now under Abbvie, is investigating the use of ChR2 to target retinal ganglion cells (RGCs) through intravitreal delivery (NCT02556736). The CAG-vector-driven intravitreal delivery of ChR2 achieved its primary endpoint with no serious adverse events reported. A total of 9/14 of patients reported adverse events (64.29%), with the most common being increased intraocular pressure (3/14). GenSight Biologics is pursuing similar goals by targeting RGC using ChrimsonR, with encouraging results, whereby the introduction of rAAV2.7m8-CAG-ChrimsonR-tdTomato intravitreally paired with stimulating medical goggles showed partial visual function recovery in a patient with non-syndromic retinitis pigmentosa (NCT03326336) [153]. Bionic Sight LLC has reported success in restoring light perception and motion detection in all 12 of its patients with RP using Chronos to target RGCs and a neural impulse producing device (NCT04278131). Their findings were dose dependent, where the highest dose group has the most vision restored [129]. Zhongmou Therapeutics similarly targeted RGCs in the same patient population with intravitreal injection of the CatCh, a variant of channelrhodopsin ChR2-L132C (NCT06292650). There are early reports of improvement in functional visual abilities, minimum light sensitivity, and overall visual performance across various simulated lighting conditions [151]. Nanoscope Therapeutics Inc. is exploring different optogenetic tools targeting ON Bipolar Cells (ON BCs), NCT04919473, NCT05417126, and NCT04945772. The Phase IIa trial STARLIGHT assessed the impact of their innovative treatment, MCO-010 (a ChR2 mutant and Chrimson), on individuals diagnosed with Stargardt disease. Patients demonstrated clinical meaningful improvements in best-corrected visual acuity. No serious adverse events were observed [149]. The same vector was used in the dose-escalated open-label safety study on 11 patients with RP, all showing vision improvement [152]. Additionally, a Phase IIb trial characterized the optogenetic therapy in patients with advanced RP, achieving its primary and key secondary endpoints with statistical significance and no serious adverse events. Nanoscope intends to submit a Biologics License Application to the FDA in the second half of 2024 [150]. In a different vein, with prospects of targeting remaining cone photoreceptor cells, the retrospective EyeConic trial NCT05294978 at University Hospital, Basel, Switzerland, is attempting to estimate proportion of IRD patients with remaining cone photoreceptors using an OCT diagnostic test. The progress in clinical trials underscores the potential of optogenetic therapy to provide improved treatment those affected by inherited retinal degenerative diseases.

## 8. Recent Advances in Targeted Gene Therapy

In recent years, significant advancements have occurred in the field gene therapy, particularly in treating inherited retinal diseases [154]. Addressing IRDs is influenced by the disease’s inheritance pattern. For autosomal recessive IRDs, such as some RP, LCA, achromatopsia, Stargardt disease, cone-rod dystrophies, and syndromic IRDs, which are characterized by a loss of function in the relevant protein, the focus lies on gene augmentation. Meanwhile, in traditionally dominantly inherited conditions, such as 20% of RP, gene therapy strategies revolve around gene suppression, sometimes combined with gene augmentation. Herein, we summarize the primary gene therapy techniques presently being investigated for the advancement of therapeutic strategies aimed at managing IRDs through preclinical studies (Table 6).

### 8.1. Retinitis Pigmentosa

Several in vivo and in vitro studies are testing the capabilities of gene editor systems in the treatment of IRD. Su and colleagues studied base editing in rd10 mice, a model of autosomal recessive RP (Pde6b mutation identified by Chang et al., 2002 [176]). Base editing is limited to single-nucleotide conversions and can correct pathogenic substitutions without generation of DNA double-strand breaks (DSBs). This strategy can target most of the identified disease causing single-nucleotide variants. However, its use is limited as it can cause a significant amount of off-target bystander edits. Additionally, the dual AAV-approach can bypass the AAV cargo limitation. These dual vector strategies have been utilized for delivering large gene supplements effectively in preclinical trials. Subretinal introduction of a split dual AAV8-ABE vector restored PDE6B expression, preserved photoreceptor cells, and restored partial retinal function (−50% rescued photopic ERG amplitude) [155]. Retinal layers were still visible 6 months after injection. However, 8.84% of bystander editing was detected near the target locus [155], outlining potential limits for its translation in clinical trials. Alternative non-viral strategies of delivery of editing constructs also include combining CRISPR/Cas9 with electroporation to enable the delivery of naked DNA to the retina [157]. This technique potentially reduces off-target effects due to the removal of bacterial elements of the plasmid and the temporary expression of Cas9. Nonetheless it also presents certain limitations, such as safety concerns, transfection efficiency (greater cell death during electroporation), and achieving adequate retinal coverage. 

In the treatment of autosomal dominant RP, Liu and colleagues harnessed allele-specific sgRNAs for T17M to target the mutant allele RHO-Ti7M in both 293 T and patient-derived iPSCs [158]. 

Examination of treated retinas revealed a lasting therapeutic impact (up to 11 months post-injection), including enhanced retinal function and preservation of photoreceptors in treated mice [158]. WGS analysis confirmed no bystander editing. Both in vitro and in vivo assessments indicated that SaCas9/17-Sg2 did not disrupt the WT RHO allele. A different strategy, a mutation-independent gene ablation and replacement system, has been used in the RHO-autosomal dominant (ad) RP humanized mouse model hRHO^C110R^/hRHO^WT^. Guided by two single-guide RNAs (sgRNAs) in a dual vector approach, approximately 60% of the target DNA underwent editing in the transduced area. Ablation and replacement methodology significantly improves photoreceptor survival and function in the humanized adRP mouse model for 12 months. In contrast, gene replacement therapy exhibited modest results in the same model [159].

In the same vein, “reduction and replacement” systems in the *Rho*-P23H knock-in mouse model of adRP have favorable results. Moreover, in ADRP human retinal explants and mutant pig models, researchers fused Cas9 to the transcriptional repressor domain, Krüppel-associated box (KRAB), and targeted this fusion protein to mutant *Rho* with a specific gRNA. This *Rho*pCRISPRi transfection resulted in 74–84% decreased promoter activity, resulting in preservation of photoreceptor cell layer thickness [84]. Both the transcriptional activators NRL and NR2E3 are involved in rod photoreceptor cell differentiation and cell homeostasis. Their modulation is shown to be a viable therapeutic strategy for RP. In three different mouse models of retinal degeneration, AAV-mediated CRISPR-Cas9 targeting Nrl in post-mitotic photoreceptors improves rod survival and preserves cone function [163].

In another approach, Nolan and colleagues proposed using CRISPR therapeutic editing to enhance aerobic glycolysis in photoreceptors over mitochondrial oxidation, making them more resilient to stress [165]. PHD2 ablation by subretinal injections of AAV8:U6-gRNAs_*PHD2* (i.e., prolyl hydroxylase domain 2)*,* targeting rod specific aerobic glycolysis via PHD-HIF (i.e., hypoxia-inducible factor) reprogramming, rescued degeneration in both recessive and dominant RP mouse models without inducing toxicity. This glycolytic reprogramming strategy confers two main advantages over mutation-specific CRISPR-based homologous repair. It could offer cost-effective treatment for diseases caused by multiple mutations and treat both dividing and non-dividing cells. 

### 8.2. Leber Congenital Amaurosis

Another group demonstrated the therapeutic potential of ABE conversion of a nonsense mutation in rd12 mice, which is a model for Rpe65-LCA [170]. Subretinal delivery via a dual-AAV serotype 9 vector appropriately induced an A to G transition in the RPE, and it was sustained 3 months after injection. In order to offset the high off-target editing rates resulting from exogenous vector administration, the group delivered a base editor and joint sgRNA ribonucleoprotein (RNP) complex for the correction of the same pathogenic variant in an identical in vivo model [166]. Both studies restored the RPE65 protein, and ERG determined the restoration resulted in significant functional recovery. However, the RNP-mediated complex approach demonstrated markedly greater editing efficiency with reduced indels compared to the classical ABE approach. Similar findings in other studies also suggest that base editing stands out as a more effective and applicable approach for disease treatment. 

This time undertaking a prime editing approach, the same group recently employed a *trans*-RNA-splicing dual AAV strategy, intravitreally injecting of two AAV8 vectors encoding N-PE and C-PE, co-injected with an additional AAV delivering pegRNA and mCherry into the rd12 mice [93]. Subretinal administration achieved 23% delivery efficiency across the RPE, with an editing efficacy of 6.4% (range: 4.1–7.4%) [93]. Editing efficiency in solely the exposed regions was estimated to be 28%, without bystander editing, indels, or off-target effects in the rd12 mouse RPE. Moreover, ERG results suggest some rescue of the disease phenotype with improved visual function [93]. LV-mediated and AAV-mediated base editing yielded higher editing efficiencies (16 ± 3% and 11 ± 5%) than AAV- delivered PE2 (6.4 ± 3%). However, no observable off-targets or undesired indels were found in rd12 mice, whereas the ABE-mediated approach showed relevant rates of bystander edits (7.7 ± 5%). Another separate investigation conducted in *rd12* mice explored the use of AAV-mediated delivery of a different prime editor to provide a method for precise correction of the Rpe65 mutation in eyes. She and colleagues delivered a dual AAV8-split PE3 (PE2 with an additional sgRNA) construct subretinally into the same mouse model [167]. Their approach yielded an editing rate of 11.4 ± 2.3% in RPE cells, with a maximal rate of 15.9%, partially restoring RPE65 expression. This intervention also enhanced photoreceptor function and viability, rescued rod and cone function, and slowed cone degeneration. 

As a proof-of-concept study, Suh et al. previously reported restauration of visual function in a rd12 mouse model using a lentiviral vector-delivered base editing strategy [171]. In seeking to enhance the on-target correction rate and reduce off-target editing, they selected an evolved ABE variant more compatible with the A6 PAM sequence to deliver to the RPE mouse [169]. NG-ABE and sgRNA-A6 were packaged into a single lentivirus vector and injected subretinally into rd12 mice. The average frequency of functionally restored alleles was (27 ± 12%). Alternative packaging into dual AAV vectors, followed by ERG testing, showed slower rescue (7 weeks in AAV vs. 3 weeks in LV delivery). To evaluate the long-term survival of cone photoreceptors, *rd12Gnat1*^−/−^-cone-function-dominant mice were injected with LV-NG-ABE-A6. ERG assessment revealed partial restoration of M-cone (36%) and S-cone (30%) function in the treated mice when compared to Gnat^−/−^ mice. Significant protection against cone loss was conferred as cones were still detected at 6 months. In another non-viral approach, empty virus-like particles (eVLPs) were used to deliver ABE RNPs to the RPE cells of rd12 mice, targeting the Rpe65 gene by subretinal injection (ABE7.10-NG-eVLPs), achieving 12% correction efficiency, with no significant bystander editing (Banskota et al., 2022) [168]. 

CRISPR–Cas9 nuclease and antisense oligonucleotides have been used to bypass one of the most common deep-intronic IRD variants splicing defect-inducing mutation in *CEP290*, another representative LCA-causing gene, in mice, primates, and human patients [177]. A different genome-editing method, termed “EDIT-101”, in which a pair of highly active and CEP290-specific saCas9 gRNAs were delivered on a human CEP290 IVS26 knock-in mouse model and in somatic primate cells, achieved a clinically efficacious rate of productive editing [172].

### 8.3. Stargardt Disease

With regards to correcting mutations in Stargardt disease, Wimmer and colleagues introduced split PE2 plasmids into HEK293 cells containing an *ABCA4* mutation to evaluate editing efficiency in vitro. Their study utilized a bioluminescence resonance energy transfer (BRAT)-based editing sensor as a measurement tool and observed corrections of up to 92% in the *ABCA4* gene [178]. In search of an optimization strategy to deliver large transgenes through AAV-based gene therapy, a similar dual AAV vector construct coined “REVeRT” by Riedmayr and colleagues was tested on a small cohort of mice, successfully reconstituting the *ABCA4* gene [173]. Similarly, McClements and colleagues administered an *ABCA4* overlapping dual vector system into Abca4^−/−^ mice [174]. Analysis via Western blotting revealed low levels of *ABCA4* expression levels throughout the neural retina of Abca4^−/−^ mice. However, treatment resulted in a decrease in bisretinoid accumulation and fundus autofluorescence levels, suggesting therapeutic potential. 

### 8.4. Clinical Trials

As previously mentioned, AAV vectors face a limitation in their payload capacity, which restricts their application in gene therapies involving larger genes. However, despite this constraint, the notable success of Luxturna studies in treating Leber congenital amaurosis, followed by clinical trials addressing conditions like RP, Leber hereditary optic neuropathy, choroideremia, Stargardt disease, and achromatopsia, all employing various serotypes with strong tropism for retinal pigment epithelium, photoreceptors, and retinal ganglion cells, has firmly established AAV vectors as the preferred choice for ocular gene therapy. 

A cumulative total of 2081 patients were enrolled across all the studies, as outlined in Table 7. The majority of these trials (52 out of 63) were classified as early phase (I/II) clinical trials, with 43 prioritizing safety as the primary outcome, while 20 emphasized treatment efficacy. Among the registered clinical trials, the targeted gene or protein varied across the studies: *RPE65* was the focus in 10 trials and post-market surveillance studies, *RPGR* in 10 studies, *ND4* in 8 studies, *CHM/REP1* in 8 studies, *CEP450* in 5 studies, *RHO* in 4 studies, *USH2A* in 3 studies, *CNGA3* in 2 studies, *CYP4V2* in 2 studies, and there was one trial each for *LCA5* and *RLBP1*.

## 9. Future Perspectives

Personalized medicine is centered on the notion that every disease, although overlapping in pathology, will be experienced uniquely by a patient based on genetic and molecular presentation. To a further extent, the treatment a patient receives can be tailored to their unique genetic and cellular background. Such an approach requires precise diagnosis, which in the care for IRDs entails identifying disease causing mutations through sequencing. In the context of gene editing or RNAi, the identification of a specific mutation will decide the optimal treatment approach. For example, a point mutation would be best addressed with a BE or PE, whereas a dominant negative mutation causing toxic protein expression would best by a “knockdown and replace” strategy, based on RNAi or CRISPR methods. Furthermore, genome size greatly influences vector choice; AAV vectors are limited to genomes lesser than 5 kb, therefore limiting its use in many IRDs. Conversely, in cases where spatial genome delivery is unachievable, optogenetics is a great alternative. Illumination provides an activation of opsins within a specific tissue area, therefore leveraging the need for tissue-specific deliveries.

Future perspectives regarding IRD treatment encompass protocols to produce retinal organoids, as they can more accurately replicate human tissue composition [179,180]. Retinal organoids do not only serve as a model for drug development but have promising applications in clinical practice. Personalized, patient-derived retinal organoids could be grown in a laboratory setting, and these patient-specific models could then be screened with multiple therapeutics. This would determine a patient’s response to a specific treatment to facilitate the optimal treatment approach [108]. The possibility to test a model that more closely resembles or is identical to human tissue will have great use in preclinical gene therapy development and future clinical practice. 

Furthermore, with the increasing landscape of artificial intelligence (AI) and machine-learning technologies, promising avenues are being explored in terms of IRD screening, diagnosis, and management [181]. Ideally, AI-based technologies will be a clinical tool that allows for the earlier detection of IRDs by accelerating diagnosis through the identification of specific disease markers that can be missed with current clinical practices. As AI continues to be studied in ophthalmology, it will gradually be integrated into clinical practice, serving as a useful tool in the diagnosis and care of IRDs. 

Finally, the landscape of optogenetics and gene therapy applications is greater; these tools have shown promising results in the treatment of non-retinal diseases as well such as glaucoma [182,183,184].

## 10. Conclusions

IRDs compromise a spectrum of clinical and genetic disorders that manifest at multiple ages, exhibiting varying severity levels and involving mutations in several genes [108]. Genetic testing for IRD-associated mutations has become standard in today’s clinical workup when IRDs are suspected, with panels capable of testing for approximately 271 known IRD-associated genes [185,186]. This is a feat that has largely been attributed to developments in next-generation sequencing but is still limited by the cost and availability of these diagnostic tests [185]. Despite access to extensive diagnostic tests, the lack of approved gene therapies can be attributed to the difficulty of translating preclinical studies into clinical therapies. This has proven to be a significant hurdle, with the most promising CRISPR and RNAi preclinical studies in model organisms failing to translate into clinical treatments. The limitation of preclinical models has led to the development of human-derived organoids, which unlike traditional cell culture, are three-dimensional multicellular constructs generated from induced-pluripotent stem cells that attempt to recapitulate human organs at a much smaller scale. Overall, numerous factors are to be considered when choosing the optimal treatment plan, based on disease characteristics, patient needs, technical resources within the clinical facility, and clinician expertise. Furthermore, with the scarce long-term safety data, use of targeted therapies should be limited to specific patient pools, based on current clinical trials. 

## Figures and Tables

**Table 2:** Summary of RNAi-based methods.

Characteristic	miRNA	siRNA	shRNA	ASOs	References
Nucleotide characteristics	20–25 bp, double-stranded RNA	21–23 bp, double-stranded RNA	19–22 bp, double-stranded RNA with a 4–11 bp hairpin loop	15–30 bp, single stranded DNA or RNA	[95,96]
Intracellular processing	Cleaved by nuclear and cytoplasmic RNase	Cleaved by cytoplasmic RNase	Expression from cDNA	No intracellular processing	[95]
Complex formed	form RISC complexes	Form RISC complexes	Form RISC complexes	Bind mRNA directly	[95]
Effect on mRNA	Interfere with translation and activate degradation	Interfere with translation and activate degradation	Interfere with translation or activate degradation	Interfere with translation, modify splicing, or activate degradation	[95]
Delivery method	Naked miRNA or vectors	Naked siRNA or vectors	Exogenous DNA expression vectors	Naked ASOs or vectors	[97,98]

Abbreviations: RNAi, RNA interference; miRNA, microRNA; siRNA, small interfering RNA; shRNA, small hairpin RNA; ASO, antisense oligonucleotides.

**Table 3:** Pros and cons of vector-based gene therapy delivery methods.

Vector Type	Advantages	Disadvantages	References
Adenovirus	Efficient transduction	Integration into host cells High immunogenicity Pathogenic virus	[105,106]
Adeno-associated viruses	Simple genome Limited integration into host genomes Multiple serotypes Efficient diffusion and transduction Non-pathogenic viruses	Small genome Small packaging capacity (<5 kilobases (kb)) Immunogenicity	[105,107,108]
Lentiviruses	Large packaging capacity Efficient transduction	Higher immunogenicity than adeno-associated viruses Mutational integration risk	[105,109]
Nanoparticles	Varying sizes to maximize packaging Chemically modifiable to alter transduction Least immunogenic Low integration risk Possible intravitreal delivery	Limited pre-clinical and clinical success in the retina	[109]

**Table 4:** Summary of preclinical phase study advances for optogenetics in the treatment of inherited retinal diseases ^a^.

Opsin Source	Optogenetic Tools	Vectors (Delivery Route)	Models	Results	References
Targeting retinal ganglion cells					
Microbial	ChR2- H134R		Heterozygous transgenic mice	Reduction of adaptation in RGCs and increased mean firing rate.	[126]
	GtCCR4	AAV7m8	*rd1* mice	GtCCR4 expression in abnormal RGCs of *rd1* mice restored light sensitivity.	[127]
	ChR-tdT	AAV2.7m8 (IV)	Macaques	Visual acuity restored to estimated 20/249 based on MEA recordings.	[128]
	Chronos	AAV2 (IV)	*rd1* mice, S334ter-3 rats (IHC safety) Cynomolgus macaques (safety)	Dose-dependent ERG response. Effective and well-tolerated across various doses and light intensities.	[129]
	ReaChR CatCh (ChR2-L132C)	AAV2 (IV)	*rd1* mice Macaques	Visual acuity restored at estimated 20/72.	[130]
	ChrimsonR	AAV2 (IV)	Macaques	Optogenetic responses remained after 1 year post transfection.	[131]
	CoChR-LC CoChR-3M	AV2 (IV) AAV2.7m8 (IV)	Opn4^−/−^ Gnat1^−/−^ Cnga3^−/−^ mice	Contrast sensitivity and visual acuity restored in ambient light and maintained 1 year post-injection.	[132]
Animal	mVChR1/ChR2/CoChR chimera (ex3mV1)	AAV2 (IV)	RCS rats	VEP recorded up to 17 months post-injection.	[133]
	MW-opsin	AAV2/2-4YF (IV)	*rd1* mice	Restoration of key aspect of natural vision with quicker response kinetics. Significant increase in VEP amplitude.	[134]
Targeting bipolar cells					
Microbial	ChCR2 mutant + Chrimson (MCO1)	AAV2 (IV)	*rd10* mice	Vision restored with dual-wavelength ChRs in ambient light. Improvement in visually guided behaviors.	[135]
Animal	MW-opsin melanopsin	AAV2.7m8	*rd1* mice	Significant increase in sensitivity than microbial alternatives. Adapted wide range of light intensities.	[136]
	Rhodopsin	AAV2.4YF (IV) AAV8.BP2 (IV) AAV2.7m8 (IV)	*rd1* mice	AAV8.BP2 and AAV2.7m8 showed greater transduction. Consistent responses with WT-like amplitude. Restored innate light avoidance.	[137]
Combined targeting of retinal ganglion cells and bipolar cells					
Microbial	CoChR-L112C	AAV2 (IV)	Opn4^−/−^ Gnat1^−/−^ Cnga3^−/−^ mice	At equivalent light intensity, the expression in RGCs yields higher visual acuity than ON BC.	[138]
Non-selective targeting					
Animal	coGHCR	AAV2 (IV)	*rd1* mice RHOP23 mice	Restored light sensitivity and object recognition in low-light environments. Protective effects against retinal degeneration.	[139]
	SWOpsin	AAV2	*rd1_KO* mice	Significantly higher correct decision rate in treated mice.	[140]
Targeting photoreceptors					
Animal	eNpHR (enhanced halorhodopsin cDNA)	AAV9 (SR)	*rcd1* dogs (*PDE6 ß*-mutant)	Partial vision restoration when targeting outer retina on larger animals.	[141]
Microbial	eNpHR	AAV2.1	*rd1* mice Rho^−/−^ Cnga3^−/−^ mice	ON, OFF, and ON/OFF light responses observed at the RGC level. Restored optomotor reflexes and light avoidance.	[69]
	Red-shifted cruxhalorhodopsin	AAV2.7m8 (IV) AAV9.7m8 (SR)	Macaques	Efficient noninvasive foveal targeting permitting robust light responses.	[142]

^a^ This table provides a summary of the preclinical phase studies published within the last five years. Additional references added to categories were novel advances deemed to be minimal. Abbreviations: ChR, channelrhodopsin; AAV, adeno-associated virus; GtCCR, light-gated cation channelrhodopsin; IV, intravitreal; CatCh, calcium-translocationg channelrhodopsin; MEA, microelectrode arrays; RGCs, retinal ganglion cells; ReaChR, red-activatable variant of CrChR2; RCS, Royal College of Surgeons; VEP, visually evoked potentials; MW, medium wavelength cone; BC, bipolar cells.

**Table 5:** Clinical trials of optogenetic therapies for vision restoration.

NCT Number (Start-End Year)	Study Phase	Target	Optogenetic Tool	Results/Notes	References
NCT05417126 (2022–2023)	Phase IIa	ON BCs Cones	vMCO-010 (ChR2 mutant + Chrimson)	Clinical meaningful improvements in best-corrected visual acuity. No serious adverse events.	[149]
NCT04945772 (2021–2024)	Phase IIb	ON BCs Cones	vMCO-010 (ChR2 mutant + Chrimson)	MCO-010 met primary and key secondary endpoints. No serious adverse events.	[150]
NCT05294978 (2021–2024)	N/A	ON BCs Cones	N/A	No results reported. OCT test to estimate IRD patients with remaining cone photoreceptors.	
NCT06292650 (2020–2029)	N/A	RGCs	CatCh (ChR2-L132C)	Improved functional visual abilities, light sensitivity, and overall visual performance in various lighting conditions.	[151]
NCT04919473 (2019–2020)	Phase I/IIa	ON BCs	vMCO-010 (ChR2 mutant + Chrimson)	Significant vision improvement in 11 patients. Treatment well tolerated.	[152]
NCT03326336 (2018–2025)	Phase I/IIa	RGCs	ChrimsonR	Partial recovery of visual function in a blind patient using light-stimulating goggles.	[153]
NCT04278131 (2018–2025)	Phase I/II	RGCs	Chronos	Dose-dependent significant vision improvement in all 12 patients.	[129]

Abbreviations: BCs, bipolar cells; RGCs, retinal ganglion cells; ChR, channelrhodopsin; calcium translocating channelrhodopsin.

**Table 6:** Summary of preclinical phase study advances for targeted gene therapy in the treatment of inherited retinal diseases ^a^.

IRD	Inheritance Mode	Genes	Animal Models	Tools	Vector	Preclinical Phase Studies	References
Retinitis pigmentosa	Autosomal recessive	*Pde6b*	*Rd10* mice	ABEs	AAV	In vivo base editing rescues photoreceptors in a mouse model of retinitis pigmentosa	[155]
				PEs	AAV	Vision rescue via unconstrained in vivo prime editing in degenerating neural retinas	[156]
				HDR		Gene Editing Preserves Visual Functions in a Mouse Model of Retinal Degeneration	[157]
		*Mertk*	RCS rat	HITI	AAV8	In vivo genome editing via CRISPR/Cas9 mediated homology-independent targeted integration	[81]
	Autosomal dominant	*RHO-T17M*	*Rho^wt/hu^* mice	BE	AAV2/8	Allele-specific gene-editing approach for vision loss restoration in RHO-associated retinitis pigmentosa	[158]
		*RHO*	hRHO^C110R^/hRHOWT	AR	AAV2/8	CRISPR genome surgery in a novel humanized model for autosomal dominant retinitis pigmentosa	[159]
			Rho*^P23H/P23H^*	AR	Dual AAV9	CRISPR/SaCas9-based gene editing rescues photoreceptor degeneration throughout a rhodopsin-associated autosomal dominant retinitis pigmentosa mouse model	[80]
			*Rho^P23H/+^* mice	mirtron	AAV	Mirtron-mediated RNA knockdown/replacement therapy for the treatment of dominant retinitis pigmentosa	[160]
			*Pro23His Pig*	KRAB	AAV	CRISPRi-Mediated Treatment of Dominant Rhodopsin-Associated Retinitis Pigmentosa	[84]
			*Rho^P23H/+^* mice	HITI	AAV8	Therapeutic homology-independent targeted integration in retina and liver	[161]
		*Nr2e3*	*Rho^P23H/+^* mice	CasMINI	AAV8	Therapeutic In Vivo Gene Editing Achieved by a Hypercompact CRISPR-Cas12f1 System Delivered with All-in-One Adeno-Associated Virus	[162]
		*Nrl*	*Rd10*, *Rho*^−/−^, RHO-P347S mice		AAV8	Nrl knockdown by AAV-delivered CRISPR/Cas9 prevents retinal degeneration in mice	[163]
			*Rd10*		AAV	Gene and mutation independent therapy via CRISPR-Cas9 mediated cellular reprogramming in rod photoreceptors	[164]
	Dominant and recessive	*PHD2/Egln1*	Rho^C110R/+^ mice (adRP) Pde6b^H620Q/H620Q^ mice (arRP)		AAV8	CRISPR editing of anti-anemia drug target rescues independent preclinical models of retinitis pigmentosa	[165]
	X-linked	*Rpgr*	*Rpgr* KO mice	HDR	AAV2/8	In Vivo CRISPR/Cas9-Mediated Genome Editing Mitigates Photoreceptor Degeneration in a Mouse Model of X-Linked Retinitis Pigmentosa	[82]
Leber congenital amaurosis	Autosomal recessive	*Rpe65 (LCA2)*	*Rd12* mice	PE3	AAVs	Application of prime editing to the correction of mutations and phenotypes in adult mice with liver and eye diseases	[93]
				ABE-RNP	LV	High-purity production and precise editing of DNA base editing ribonucleoproteins	[166]
				PE2	AAV	Dual-AAV split prime editor corrects the mutation and phenotype in mice with inherited retinal degeneration	[167]
				ABEs	eVLPs	Engineered virus-like particles for efficient in vivo delivery of therapeutic proteins	[168]
				NG-ABE	LV	In vivo base editing rescues cone photoreceptors in a mouse model of early-onset inherited retinal degeneration	[169]
				ABEs	AAV	Visual function restoration in a mouse model of Leber congenital amaurosis via therapeutic base editing	[170]
			*Rd12* mice *rd12 Gnat1*^−/−^ mice	ABEs	LV	Restoration of visual function in adult mice with an inherited retinal disease via adenine base editing	[171]
		*CEP290*	HuCEP290 knock-in mice and monkeys	EDIT-101	AAV	Development of a gene-editing approach to restore vision loss in Leber congenital amaurosis type 10	[172]
Stargardt disease	Autosomal recessive	*ABCA4*	*Abca4^−/−^ Rdh8^−/−^* mice	REVeRT	AAV	mRNA trans-splicing dual AAV vectors for (epi)genome editing and gene therapy	[173]
			*Abca4^−/−^* mice		AAV	An AAV Dual Vector Strategy Ameliorates the Stargardt Phenotype in Adult Abca4^−/−^ Mice	[174]
Cone-rod dystrophy 6	Autosomal dominant	*GUCY2D*	RetGC1 (hR838S, hWT) mouse	AR	AAV	Development of an AAV-CRISPR-Cas9-based treatment for dominant cone-rod dystrophy 6	[112]
X-linked juvenile retinoschisis	X-linked	*RS1*	BALB/c-strain mice	HITI	SMNP	Dual Supramolecular Nanoparticle Vectors Enable CRISPR/Cas9-Mediated Knockin of Retinoschisin 1 Gene—A Potential Nonviral Therapeutic Solution for X-Linked Juvenile Retinoschisis	[175]

^a^ This table provides a summary of the preclinical phase studies published within the last five years. Additional references were added to categories where novel advances were deemed to be minimal. Abbreviations: ABEs, adenine base editors; PE, prime editors; HDR, homology-directed repair; HITI, homology-independent targeted integration; BE, base editors; AAV, adeno-associated virus; LV, lentivirus; AR, ablation and replacement.

**Table 7:** Clinical trials of targeted gene therapy for vision restoration ^a^.

NCT Number	Gene	Vector	Interventions	Study Title	Phases
Achromatopsia					
NCT02935517	*CNGA3*	AAV8	AGTC-402	Safety and Efficacy Trial of AAV Gene Therapy in Patients With CNGA3 Achromatopsia (A Clarity Clinical Trial)	Phase 1, Phase 2
NCT02610582	*CNGA3*	AAV2	rAAV.hCNGA3	Safety and Efficacy of rAAV.hCNGA3 Gene Therapy in Patients With CNGA3-linked Achromatopsia	Phase 1, Phase 2
NCT02599922	*CNGB3*	AAV2	rAAV2tYF-PR1.7-hCNGB3	Safety and Efficacy Trial of AAV Gene Therapy in Patients With CNGB3 Achromatopsia (A Clarity Clinical Trial)	Phase 1, Phase 2
NCT03001310	*CNGB3*	AAV2/8	AAV2/8-hCARp.hCNGB3	Gene Therapy for Achromatopsia (CNGB3) (CNGB3)	Phase 1, Phase 2
Bietti Crystalline Dystrophy					
NCT05399069	*CYP4V2*	AAV8	VGR-R01	Safety and Tolerability of VGR-R01 in Patients With Bietti Crystalline Dystrophy	Early Phase 1
NCT04722107	*CYP4V2*	AAV2/8	rAAV2/8-hCYP4V2	Safety Study of rAAV2/8-hCYP4V2 in Patients With Bietti’s Crystalline Dystrophy (BCD)	Early Phase 1
Choroideremia					
NCT03507686	*CHM/REP1*	AAV2	BIIB111 (AAV2-REP1)	A Safety Study of Retinal Gene Therapy for Choroideremia With Administration of BIIB111	Phase 2
NCT03496012	*CHM/REP1*	AAV2	BIIB111 (AAV2-REP1)	Efficacy and Safety of BIIB111 for the Treatment of Choroideremia	Phase 3
NCT02553135	*CHM/REP1*	AAV2	AAV2-REP1	Choroideremia Gene Therapy Clinical Trial	Phase 2
NCT02341807	*CHM/REP1*	AAV2	AAV2-hCHM	Safety and Dose-escalation Study of AAV2-hCHM in Participants With CHM (Choroideremia) Gene Mutations	Phase 1, Phase 2
NCT02671539	*CHM/REP1*	AAV2	rAAV2.REP1	THOR—Tübingen Choroideremia Gene Therapy Trial	Phase 2
NCT02077361	*CHM/REP1*	AAV2	rAAV2.REP1	An Open Label Clinical Trial of Retinal Gene Therapy for Choroideremia	Phase 1, Phase 2
NCT02407678	*CHM/REP1*	AAV2	AAV-mediated REP1 gene replacement	REP1 Gene Replacement Therapy for Choroideremia	Phase 2
NCT01461213	*CHM/REP1*	AAV2	rAAV2.REP1	Gene Therapy for Blindness Caused by Choroideremia	Phase 1, Phase 2
Leber Congenital Amaurosis					
NCT03920007	*GLUCY2D*	AAV5	ATSN-101	Study of Subretinally Injected ATSN-101 Administered in Patients With Leber Congenital Amaurosis Caused by Biallelic Mutations in GUCY2D	Phase 1, Phase 2
NCT00749957	*RPE65*	AAV2	rAAV2-CB-hRPE65	Phase 1/2 Safety and Efficacy Study of AAV-RPE65 Vector to Treat Leber Congenital Amaurosis	Phase 1, Phase 2
NCT00821340	*RPE65*	AAV2	rAAV2-hRPE65	Clinical Trial of Gene Therapy for Leber Congenital Amaurosis Caused by RPE65 Mutations	Phase 1
NCT05906953	*RPE65*	AAV2	HG004	Safety and Efficacy Trial of HG004 for Leber Congenital Amaurosis Related to Rpe65 Gene Mutations (STAR)	Phase 1, Phase 2
NCT02781480	*RPE65*	AAV2/5	AAV RPE65	Clinical Trial of Gene Therapy for the Treatment of Leber Congenital Amaurosis (LCA)	Phase 1, Phase 2
NCT01496040	*RPE65*	AAV4	rAAV2/4.hRPE65	Clinical Gene Therapy Protocol for the Treatment of Retinal Dystrophy Caused by Defects in RPE65	Phase 1, Phase 2
NCT00999609	*RPE65*	AAV2	AAV2-hRPE65v2,voretigene neparvovec-rzyl	Safety and Efficacy Study in Subjects With Leber Congenital Amaurosis	Phase 3
NCT00516477	*RPE65*	AAV2	AAV2-hRPE65v2 (Luxterna; voretigene neparvovec-rzyl)	Safety Study in Subjects With Leber Congenital Amaurosis	Phase 1
NCT00643747	*RPE65*	AAV2	tgAAG76 (rAAV2/2.hRPE65p.hRPE65)	Safety Study of RPE65 Gene Therapy to Treat Leber Congenital Amaurosis	Phase 1, Phase 2
NCT00481546	*RPE65*	AAV2	rAAV2-CBSB-hRPE65	Phase I Trial of Gene Vector to Patients With Retinal Disease Due to RPE65 Mutations	Phase 1
NCT06088992	*RPE65*	AAV9	HG004	Leber Congenital Amaurosis Inherited Blindness of Gene Therapy Trial(LIGHT)	Early Phase 1
NCT03872479	*IVS26/CEP290*	AAV5	EDIT-101	Single Ascending Dose Study in Participants With LCA10	Phase 1, Phase 2
NCT03913143	*CEP290*	ASO	QR-110 (sepofarsen)	A Study to Evaluate Efficacy, Safety, Tolerability and Exposure After a Repeat-dose of Sepofarsen (QR-110) in LCA10 (ILLUMINATE)	Phase 2, Phase 3
NCT03913130	*CEP290*	ASO	QR-110 (sepofarsen)	Extension Study to Study PQ-110-001 (NCT03140969)	Phase 1, Phase 2
NCT04855045	*CEP290*	ASO	QR-110 (sepofarsen)	An Open-label, Dose Escalation and Double-masked, Randomized, Controlled Trial Evaluating Safety and Tolerability of Sepofarsen in Children (<8 Years of Age) With LCA10 Caused by Mutations in the CEP290 Gene.	Phase 2, Phase 3
NCT03140969	*CEP290*	ASO	QR-110 (sepofarsen)	Study to Evaluate QR-110 in Leber’s Congenital Amaurosis (LCA) Due to the c.2991 + 1655A > G Mutation (p.Cys998X) in the CEP290 Gene	Phase 1, Phase 2
NCT05616793	*LCA5*	AAV8	AAV8.hLCA5	Safety and Tolerability Subretinal OPGx-001 for LCA5-Associated Inherited Retinal Degeneration (LCA5-IRD)	Phase 1, Phase 2
Leber Hereditary Optic Neuropathy					
NCT01267422	*MT-ND4*	AAV2	rAAV2-ND4	Safety and Efficacy Study of rAAV2-ND4 Treatment of Leber Hereditary Optic Neuropathy (LHON)	NA
NCT02161380	*MT-ND4*	AAV2	scAAV2-P1ND4v2	Safety Study of an Adeno-associated Virus Vector for Gene Therapy of Leber’s Hereditary Optic Neuropathy	Phase 1
NCT03406104	*MT-ND4*	AAV2	GS010	RESCUE and REVERSE Long-term Follow-up	Phase 3
NCT02064569	*MT-ND4*	AAV2	GS010	Safety Evaluation of Gene Therapy in Leber Hereditary Optic Neuropathy (LHON) Patients	Phase 1, Phase 2
NCT02652780	*MT-ND4 (G11778A)*	AAV2	GS010	Efficacy Study of GS010 for Treatment of Vision Loss From 7 Months to 1 Year From Onset in LHON Due to the ND4 Mutation (REVERSE)	Phase 3
NCT02652767	*MT-ND4 (G11778A)*	AAV2	GS010	Efficacy Study of GS010 for the Treatment of Vision Loss up to 6 Months From Onset in LHON Due to the ND4 Mutation	Phase 3
NCT03293524	*MT-ND4*	AAV2	GS010	Efficacy & Safety Study of Bilateral IVT Injection of GS010 in LHON Subjects Due to the ND4 Mutation for up to 1 Year	Phase 3
NCT03153293	*MT-ND4*	AAV2	rAAV2-ND4	A Single Intravitreal Injection of rAAV2-ND4 for the Treatment of Leber’s Hereditary Optic Neuropathy	Phase 2, Phase 3
Retinitis Pigmentosa					
NCT03328130	*PDE6B*	AAV2/5	AAV2/5-hPDE6B	Safety and Efficacy Study in Patients With Retinitis Pigmentosa Due to Mutations in PDE6B Gene	Phase 1, Phase 2
NCT01482195	*MERTK*	AAV2	rAAV2-VMD2-hMERTK	Trial of Subretinal Injection of (rAAV2-VMD2-hMERTK)	Phase 1
NCT03374657	*RLBP1*	AAV8	CPK850	A First-in-human, Proof of Concept Study of CPK850 in Patients With RLBP1 Retinitis Pigmentosa	Phase 1, Phase 2
NCT06388200	*RHO/NR2E3*	AAV	OCU400-301	A Phase 3 Study Of OCU400 Gene Therapy for the Treatment Of Retinitis Pigmentosa	Phase 3
NCT05805007	*RHO*	AAV	ZVS203e	Safety and Tolerability Study of Gene Editing Drug ZVS203e in Participants With Retinitis Pigmentosa	Early Phase 1
NCT04611503	*PDE6A*	AAV	rAAV.hPDE6A	PDE6A Gene Therapy for Retinitis Pigmentosa	Phase 1, Phase 2
NCT06291935	*CNGA1*	AAV2	VG901 (AAV2.NN-CNGA1)	Safety and Tolerability of Intravitreal Administration of VG901 in Patients With Retinitis Pigmentosa Due to Mutations in the CNGA1 Gene	Phase 1
NCT04123626	*P23H (RHO)*	ASO	QR-1123	A Study to Evaluate the Safety and Tolerability of QR-1123 in Subjects With Autosomal Dominant Retinitis Pigmentosa Due to the P23H Mutation in the RHO Gene	Phase 1, Phase 2
NCT05176717	*USH2A (exon 3)*	ASO	Ultevursen (QR-421a)	Study to Evaluate the Efficacy Safety and Tolerability of QR-421a in Subjects With RP Due to Mutations in Exon 13 of the USH2A Gene With Early to Moderate Vision Loss (Celeste)	Phase 2, Phase 3
NCT05158296	*USH2A (exon 3)*	ASO	Ultevursen (QR-421a)	Study to Evaluate the Efficacy Safety and Tolerability of Ultevursen in Subjects With RP Due to Mutations in Exon 13 of the USH2A Gene (Sirius)	Phase 2, Phase 3
NCT03780257	*USH2A (exon 3)*	ASO	Ultevursen (QR-421a)	Study to Evaluate Safety and Tolerability of QR-421a in Subjects With RP Due to Mutations in Exon 13 of the USH2A Gene	Phase 1, Phase 2
NCT04517149	*RPGR*	(AAV) R100	4D-125	4D-125 in Patients With X-Linked Retinitis Pigmentosa (XLRP)	Phase 1, Phase 2
NCT06333249	*RPGR*	AAV2	AGTC-501 (rAAV2tYF-GRK1-RPGR)	A Study Comparing Two Doses of AGTC-501 in Male Subjects With X-linked Retinitis Pigmentosa Caused by RPGR Mutations (SKYLINE)	Phase 2
NCT06275620	*RPGR*	AAV2	AGTC-501 (rAAV2tYF-GRK1-RPGR)	A Study Comparing Two Doses of AGTC-501 in Male Participants With X-linked Retinitis Pigmentosa Caused by RPGR Mutations (DAWN)	Phase 2
NCT04850118	*RPGR*	AAV2	rAAV2tYF-GRK1-hRPGRco	A Clinical Trial Evaluating the Safety and Efficacy of a Single Subretinal Injection of AGTC-501 in Participants With XLRP	Phase 2, Phase 3
NCT03316560	*RPGR*	AAV2	rAAV2tYF-GRK1-RPGR	Safety and Efficacy of rAAV2tYF-GRK1-RPGR in Subjects With X-linked Retinitis Pigmentosa Caused by RPGR Mutations	Phase 1, Phase 2
NCT03116113	*RPGR*	AAV8	BIIB112 (AAV8-RPGR)	A Clinical Trial of Retinal Gene Therapy for X-linked Retinitis Pigmentosa Using BIIB112	Phase 1, Phase 2
NCT05874310	*RPGR*	AAV	FT-002	Gene Therapy for Subjects With RPGR Mutation-associated X-linked Retinitis Pigmentosa	Early Phase 1
NCT04794101	*RPGR*	AAV5	AAV5-hRKp.RPGR	Follow-up Gene Therapy Trial for the Treatment of X-linked Retinitis Pigmentosa Associated With Variants in the RPGR Gene	Phase 3
NCT04671433	*RPGR*	AAV5	AAV5-hRKp.RPGR	Gene Therapy Trial for the Treatment of X-linked Retinitis Pigmentosa Associated With Variants in the RPGR Gene	Phase 3
NCT03252847	*RPGR*	AAV2/5	AAV2/5-RPGR	Gene Therapy for X-linked Retinitis Pigmentosa (XLRP)—Retinitis Pigmentosa GTPase Regulator (RPGR)	Phase 1, Phase 2
Stargardt Disease 1					
NCT06300476	*ABCA4*	AAV	JWK006	Safety and Efficacy of a Single Subretinal Injection of JWK006 Gene Therapy in Subjects With Stargardt Disease(STGD1)	Phase 1, Phase 2
X-Linked Retinoschisis					
NCT06289452	*RS1*	AAV8	IVB102	Safety and Efficacy Study of IVB102 Injection in Subjects With X-linked Retinoschisis	Early Phase 1

^a^ Clinical trials summary obtained from ClinicalTrials.gov. The table lists ongoing clinical trials for the treatment of inherited retinal diseases using targeted gene therapy.
